# Obese patients with CEAP (clinical, etiology, anatomy, pathophysiology) C2 and C3 disease show enhanced symptom improvement after endovenous thermal ablation

**DOI:** 10.1016/j.jvsv.2024.101873

**Published:** 2024-03-19

**Authors:** Zachary R. Zottola, Joshua T. Geiger, Geena E. Choo, Baqir J. Kedwai, Mark D. Balceniuk, Jennifer L. Ellis, Adam J. Doyle, Karina A. Newhall

**Affiliations:** Division of Vascular Surgery, Department of Surgery, University of Rochester Medical Center, Rochester, NY

**Keywords:** Laser therapy, Obesity, Patient-reported outcome measures, Radiofrequency ablation, Venous insufficiency

## Abstract

**Objective:**

Endovenous thermal ablation (EVTA) is a prevalent treatment option for patients with severe venous disease. However, the decision to intervene for patients with less severe disease (CEAP [clinical, etiology, anatomy, pathophysiology] C2 and C3) is less clear and becomes further complicated for patients with obesity, a pathology known to increase venous disease symptom severity. Therefore, the objective of this study was to use the Society for Vascular Surgery Vascular Quality Initiative database to evaluate outcomes after EVTA in obese patients with CEAP C2 and C3 venous insufficiency.

**Methods:**

Using the Society for Vascular Surgery Vascular Quality Initiative database, we retrospectively analyzed the initial procedure of all patients with a CEAP clinical class of C2 or C3 who underwent EVTA from January 2015 to December 2021. Patients were grouped by obesity, defined as a body mass index of ≥30 kg/m^2^. The primary outcome was the change in venous clinical severity score (VCSS) from the procedure to the patient's initial follow-up. The secondary outcomes included the change in patient-reported outcomes at follow-up via the HASTI (heaviness, achiness, swelling, throbbing, itching) score, incidence of follow-up complications, and recanalization of treated veins. The change in the VCSS and HASTI score were analyzed using Student *t* tests, and complications and recanalization were assessed using the Fisher exact test. Significant outcomes were confirmed by multiple variable logistic regression. The remaining significant variables were then analyzed, with obesity categorized using the World Health Organization classification system to analyze how increasing obesity levels affect outcomes.

**Results:**

There were 8146 limbs that met the inclusion criteria, of which 5183 (63.6%) were classified as nonobese and 2963 (36.4%) as obese. Obesity showed no impact on improvement in the VCSS (−3.29 vs −3.35; *P* = .408). Obesity was found to be associated with a larger improvement in overall symptoms, as evidence by a greater improvement in the HASTI score (−7.24 vs −6.62; *P* < .001). Obese limbs showed a higher incidence of superficial phlebitis (1.5% vs 0.7%; *P* = .001), but no difference was found in recanalization or any other complication.

**Conclusions:**

These data suggest that obese patients with CEAP clinical class C2 or C3 experience greater improvement in their perceived symptoms after EVTA with little difference in clinical improvement and complications compared with nonobese patients. Although obesity has been associated with increased severity of venous disease symptoms, obese patients are able to derive significant relief after treatment during the short term and may experience greater relief of symptoms than nonobese patients when treated at more mild disease presentations.

**Article Highlights**

• **Type of Research:** A retrospective review of prospectively collected Vascular Quality Initiative data.
• **Key Findings:** Obese limbs with CEAP (clinical, etiology, anatomy, pathophysiology) C2 and C3 venous disease demonstrate no difference in venous clinical severity score improvement (change, −3.29 vs −3.35; *P* = .408) compared with nonobese limbs but do show greater perceived symptom improvement via a greater reduction in their HASTI (heaviness, achiness, swelling, throbbing, itching) score (−7.24 vs −6.62; *P* = .001) following endovenous thermal ablation.
• **Take Home Message:** Obese patients experience greater short-term symptom relief after endovenous thermal ablation than nonobese patients in the setting of more mild disease presentations.

Obesity represents one of the fastest growing concerns in the United States due to its association with numerous medical comorbidities.^1^ Along with this growing concern, is the concern for obesity's impact on lower limb chronic venous insufficiency (CVI). It has been shown there is both an increased prevalence of CVI in individuals with a higher body mass index (BMI) and that obese patients are more likely to present with symptomatic venous disease than are nonobese patients.^2^ Additional studies have also been able to establish a positive association between an increasing BMI and the severity of venous disease as assessed by the CEAP (clinical, etiology, anatomy, pathophysiology) classification and the venous clinical severity score (VCSS).^3^, ^4^, ^5^, ^6^ As such, a growing obese population raises concerns for an increase in symptomatic venous disease as well.

To combat venous disease, endovenous thermal ablation (EVTA) techniques, such as endovenous radiofrequency ablation (RFA) and endovenous laser ablation (EVLA) have emerged as prevalent treatment options due to their minimally invasive nature and their ability to be performed in an outpatient setting compared with other surgical repair options.^7^, ^8^, ^9^, ^10^ Although intervention to treat more severe venous disease, such as CEAP clinical classes C4, C5, and C6, is indicated to treat and prevent progression of ulceration and epithelial damage, there is a lack of literature and definitive recommendations for EVTA in patients with more mild venous disease. This is further complicated for patients with obesity as there is a similar lack of literature concerning the effect of obesity on EVTA outcomes. With a growing discussion around early endovenous approaches to CVI, it is worth exploring obesity's impact on treatment in these patients,^11^^,^^12^ a cohort frequently encountered in robust venous practices. Therefore, the objective of this study was to use the Society for Vascular Surgery Vascular Quality Initiative (VQI) to evaluate outcomes after EVTA in obese patients compared with nonobese patients with a lower CEAP clinical class (C2 and C3).

## Methods

### Study database

This study analyzes the VQI, an international, prospectively maintained dataset encompassing 14 vascular surgery procedures performed throughout the United States and Canada.^13^ A retrospective review of the U.S. VQI Varicose Vein module from January 2015 to December 2021 was performed. The University of Rochester Medical Center institutional review board approved this study and waived the requirement for patient consent (approval no. STUDY00007618).

### Study population

All the patients were analyzed by individual limb. Eligible limbs were identified based on a CEAP clinical class of C2 or C3 to represent more mild venous disease. Although CEAP class is meant for use as a descriptive classification system and is not approved for use as a severity score, the CEAP class, instead of the VCSS, was used to identify the study sample as it is the most used classifier of CVI and provides a more standard, universal description of the studied population.^14^ C2 and C3 were chosen to represent more mild venous disease because they represent stages of CVI that justify intervention but do not include superficial epithelial damage such as in classes C4, C5, and C6 (Supplementary Table I, online only). Class C0 and C1 limbs were excluded because these patients infrequently undergo intervention. All limbs undergoing EVTA (either RFA or EVLA) from 2015 to 2021 were included in the analysis. Patients who underwent any chemical ablation treatment were not included as data describing the type and specific agents used in these procedures is limited in the VQI database. Limbs were excluded if data were missing for the CEAP class, VCSS, or patient height or weight. Limbs with missing long-term follow-up (LTF) data or an underweight patient BMI were also excluded. Only the initial postprocedure follow-up examination was used to assess the follow-up outcomes for this analysis, with patients included if the initial follow-up visit was recorded as <3 months, in an attempt to target the true first follow-up appointment and control for missing data.

The limbs were initially grouped into obese and nonobese categories based on a patient BMI of ≥30 kg/m^2^. Additional analysis was performed with the limbs grouped into normal weight (BMI, <25 kg/m^2^), overweight (BMI, 25-29.9 kg/m^2^), obese class 1 (BMI, 30-34.9 kg/m^2^), obese class 2 (BMI, 35-39.9 kg/m^2^), and obese class 3 (BMI, ≥40 kg/m^2^) based on the World Health Organization (WHO) classification of obesity.

### Limb characteristics

Patient demographics, procedural data, and preprocedural patient-reported outcomes (PROs) were collected to analyze the differences between the limbs of obese and nonobese patients. The demographics included age, BMI, gender, race, a history of phlebitis, a history of deep vein thrombosis (DVT), a history of prior vein treatment, the presence of venous thrombus (either deep or superficial) in the intervention leg, preprocedural CEAP class, and preprocedural VCSS. The procedure variables included the maximum vein diameter, vein length treated, number of veins treated, long-term follow-up anticoagulation use, the use of a concomitant surgery (ie, stripping, high ligation with stripping, stab phlebectomy, transillumination phlebectomy, or open ligation), location of the primary treated vein, and follow-up compression use. Preprocedural PROs, a group of symptoms graded on a severity score of 0 to 5, with 5 representing the greatest severity, included perceived heaviness, achiness, swelling, throbbing, itching, appearance, and work impact. A composite score termed the HASTI (heaviness, achiness, swelling, throbbing, itching) score was also created by summing the individual scores of heaviness, achiness, swelling, throbbing, and itching. The HASTI score is a previously validated symptom assessment tool that was derived from the VEINES-QOL (venous insufficiency epidemiological and economic study–quality of life) survey^15^ and has been used in previous work to assess symptom improvement after venous treatment.^16^^,^^17^

### Outcomes

The primary outcome was the change in the VCSS from before the procedure to the initial follow-up. Because CEAP has been cited as a descriptive modality and not suitable for determining clinical improvement, the VCSS was chosen as the primary outcome.^14^^,^^18^ However, the CEAP class has been previously used in studies to show clinical improvement and, as such, was provided as a descriptive variable for additional data comparison. The secondary outcomes included the change in HASTI score from before the procedure to the initial follow-up and the change in individual PROs. The secondary outcomes also included the incidence of follow-up procedural complications as defined by the VQI (ie, bleeding requiring intervention, blistering, DVT, hematoma, paresthesia, pigmentation, superficial phlebitis, treatment-induced ulcer, wound infection, endothermal heat-induced thrombosis [EHIT]) and early vein recanalization.

### Statistical analysis

The Student *t* test for continuous variables and the Fisher exact test for categorical variables were used to identify demographic and procedural differences between the two groups. To confirm that both obese and nonobese limbs experience significant improvement after treatment, the preprocedural VCSS and PROs were compared with the initial follow-up values using the paired Student *t* test. New delta variables (change in VCSS, HASTI, and each PRO) were then created by subtracting the initial follow-up value from the preprocedural value for the individual limbs. These new variables represent the postprocedural changes for a given outcome in that a negative number is a beneficial decrease and a positive number is a harmful increase. These were then compared between the obese and nonobese limbs using the Student *t* test, and the incidence of procedural complications was compared using the Fisher exact test.

Outcomes found to be significant on univariate analysis were further analyzed using separate multiple logistic regressions to account for baseline differences between groups. For continuous outcomes (ie, VCSS, HASTI score, PROs), this was done by analyzing a variable called “postprocedural improvement,” a binomial outcome created for each dependent variable of interest, where postprocedural improvement was coded as “1” for any beneficial decrease in the value of an outcome and as “0” for any harmful increase. These new variables were then used to render odds ratios (ORs) in subsequent multiple logistic regressions accounting for all collected demographic and procedural variables described previously, including the use of concomitant surgery to account for variations in procedure and preprocedural CEAP class to account for differences in C2 and C3 frequency among groups. Multiple logistic regressions were also conducted for complication outcomes.

Following the initial multivariate analysis, all significant outcomes were then analyzed, with the limbs categorized into five WHO BMI categories. Individual multiple logistic regressions, accounting for the collected demographic and procedural data, were conducted based on this categorical classification, to analyze the degree to which increasing obesity levels would affect each outcome.

Univariable logistic regression of each covariate, followed by backward elimination in multiple logistic regression, was used to reduce the chance of overfitting models. Statistical significance is reported with a *P* value of ≤ .05 for all analyses. Statistical analysis was performed using Stata, version 17.0 (StataCorp).

## Results

A total of 8146 limbs met the inclusion criteria, of which 5183 (63.6%) were classified as nonobese and 2963 (36.4%) as obese (Table I). The median time to follow-up was 20 days. Obese limbs were found in patients who were older (54.2 years vs 53.5 years; *P* = .038) and more likely to have a history of phlebitis (10.7% vs 8.9%; *P* = .010), a history of DVT (5.9% vs 3.7%; *P* < .001), and a higher rate of preprocedural thrombus present at intervention (4.9% vs 3.5%; *P* = .002). Obese limbs were less likely to be found in women (73.2% vs 76.9%; *P* < .001) and less likely to have undergone a prior vein treatment (21.7% vs 24.4%; *P* < .001). No difference was found for race. Obese limbs had a higher average VCSS (6.80 vs 6.43; *P* < .001) and presented with more limbs classified as CEAP C3 compared with nonobese limbs (60.1% vs 44.6%; *P* < .001). Obese limbs presented with larger maximum vein diameters (8.10 mm vs 7.59 mm; *P* < .001), had greater lengths of vein treated (36.7 mm vs 34.4 mm; *P* < .001), were more likely to receive anticoagulation therapy at follow-up (6.0% vs 3.9%; *P* < .001), and were more likely to use compression daily after the procedure (41.2% vs 37.1%; *P* < .001). Obese limbs had a fewer number of veins treated (1.99 vs 2.06; *P* = .001) and were less likely to undergo concomitant surgery (45.2% vs 52.4%; *P* < .001). No difference was found in the location of the vein treated. Obese patients, on average, reported higher scores for every PRO, except for the reported limb appearance (Table I).

**Table I:** Patient and limb characteristics compared between obese and nonobese limbs

Characteristic	Obese		*P* value
	No (n = 5183)	Yes (n = 2963)	
Demographics			
Age, years	53.49 ± 14.13	54.15 ± 12.88	.038
BMI, kg/m^2^	25.07 ± 2.88	35.72 ± 5.23	<.001
Female gender	3983 (76.9)	2168 (73.2)	<.001
Non-White race	981 (18.9)	547 (18.5)	.616
History of phlebitis	460 (8.9)	316 (10.7)	.010
History of DVT	193 (3.7)	175 (5.9)	<.001
History of vein treatment	1261 (24.4)	642 (21.7)	.006
Preoperative thrombus	181 (3.5)	145 (4.9)	.002
VCSS	6.43 ± 2.00	6.80 ± 2.01	<.001
CEAP class			<.001
C2	2872 (55.4)	1182 (39.9)	
C3	2311 (44.6)	1781 (60.1)	
Procedure variables			
Maximum diameter, mm	7.59 ± 3.36	8.10 ± 3.50	<.001
Vein length, mm	34.37 ± 15.81	36.74 ± 16.53	<.001
No. of veins treated	2.06 ± 1.13	1.99 ± 1.15	.001
Follow-up anticoagulation use	205 (3.9)	179 (6.0)	<.001
Concomitant surgery	2714 (52.4)	1339 (45.2)	<.001
Location of primary vein treated			.204
Truncal	5080 (98.0)	2916 (98.4)	
Truncal, recanalized	60 (1.2)	33 (1.1)	
Cluster	26 (0.5)	6 (0.2)	
Perforator	17 (0.3)	8 (0.3)	
Follow-up compression use			<.001
No	1320 (25.5)	662 (22.3)	
Intermittent	1373 (26.5)	795 (26.8)	
Most days	566 (10.9)	284 (9.6)	
Daily	1924 (37.1)	1222 (41.2)	
PROs			
HASTI score	9.21 ± 4.85	10.32 ± 5.21	<.001
Heaviness	2.06 ± 1.46	2.21 ± 1.51	<.001
Achiness	2.51 ± 1.30	2.68 ± 1.33	<.001
Swelling	1.91 ± 1.51	2.33 ± 1.57	<.001
Throbbing	1.64 ± 1.43	1.87 ± 1.51	<.001
Itching	1.09 ± 1.37	1.24 ± 1.45	<.001
Appearance	2.51 ± 1.15	2.41 ± 1.20	<.001
Work impact	1.83 ± 1.24	1.99 ± 1.28	<.001

*BMI,* Body mass index; *CEAP,* clinical, etiology, anatomy, pathophysiology; *DVT,* deep vein thrombosis; *HASTI,* heaviness, achiness, swelling, throbbing, itching; *PROs,* patient-reported outcomes; *VCSS,* venous clinical severity score.

Data presented as mean ± standard deviation (continuous variables) or number (%; categorical variables).

**Fig 1:**
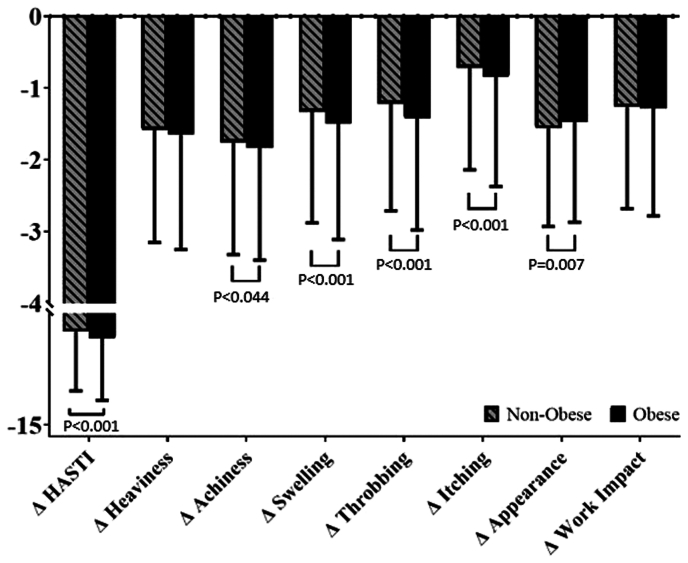
Univariate analysis of the change in patient-reported outcomes (PROs) between obese and nonobese cohorts as assessed by the Student *t* test. Negative values represent a beneficial reduction in the PRO from before the procedure to the initial follow-up visit. All significant *P* values are labeled within the graph. HASTI, heaviness, achiness, swelling, throbbing, itching.

**Fig 2:**
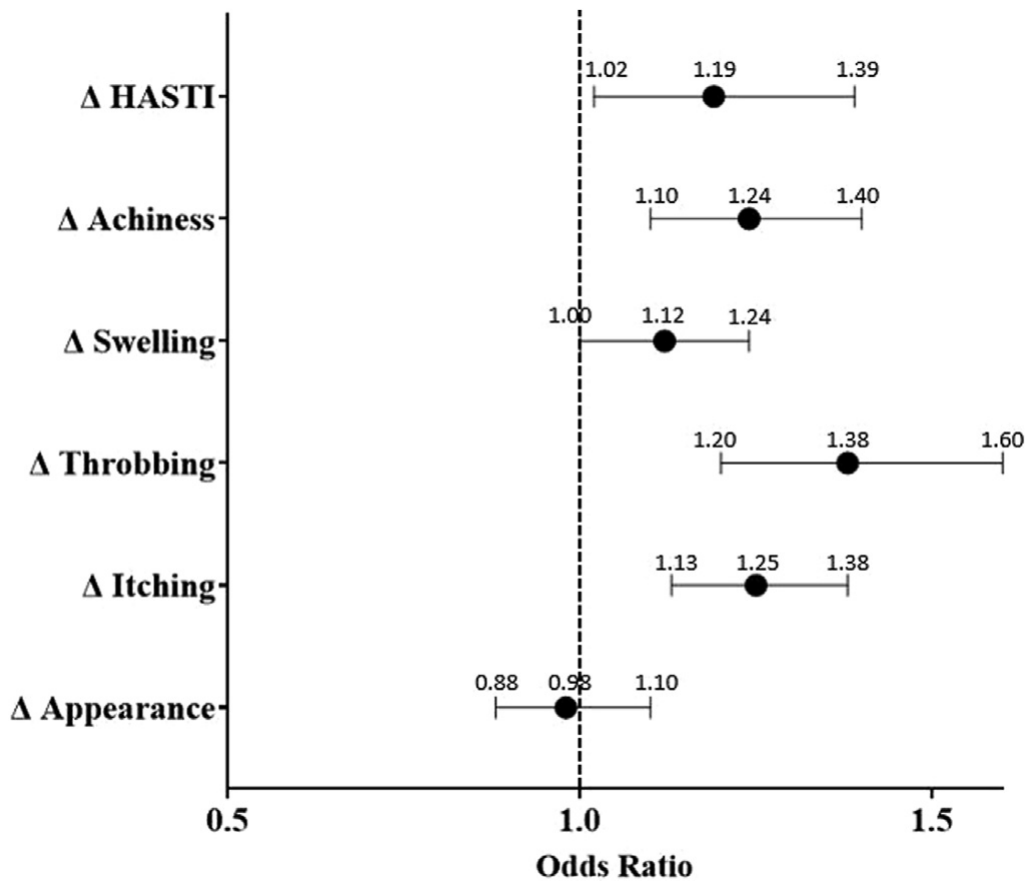
Odds ratios (ORs) and 95% confidence intervals (CIs) of obesity's impact on the postprocedure improvement for patient-reported outcomes (PROs) found to be significant on previous univariate analysis.

A comparison of the preprocedural and initial follow-up VCSSs and PROs shows that both obese and nonobese limbs with C2 and C3 disease experience significant clinical and symptomatic improvement after EVTA (Supplementary Table II, online only). The percentage of limbs in each CEAP category postprocedurally is presented in Table II. No association was found between the obese and nonobese limbs for the change in the VCSS (Table III). Obese limbs showed a significantly greater reduction in the HASTI score (−7.24 vs −6.62; *P* = .001) and the individual categories of achiness (−1.83 vs −1.76; *P* = .044), swelling (−1.49 vs −1.33; *P* < .001), throbbing (−1.42 vs −1.22; *P* < .001), and itching (−0.84 vs −0.72; *P* < .001; Fig 1). Obese limbs demonstrated less reduction in appearance (−1.47 vs −1.56; *P* = .007). No association was found between the two groups for the change in heaviness or work impact. Multivariate logistic regressions using the defined “postprocedural improvement” variables confirmed the VCSS to not be significant. Greater improvement in the HASTI score (OR, 1.19; *P* = .026) and the individual categories of achiness (OR, 1.24; *P* = .001), swelling (OR, 1.12; *P* < .043), throbbing (OR, 1.38; *P* < .001), and itching (OR, 1.25; *P* < .001) remained statistically significant for the obese limbs (Table III; Fig 2). The reduced improvement in the reported limb appearance did not remain significant.

**Table II:** Description of CEAP (clinical, etiology, anatomy, pathophysiology) clinical class frequency before and after procedure

CEAP class	Nonobese (n = 5183)		Obese (n = 2963)	
	Preoperative	LTF	Preoperative	LTF
C0	0 (0)	545 (10.5)	0 (0)	223 (7.5)
C1	0 (0)	1997 (38.5)	0 (0)	988 (33.3)
C2	2872 (55.4)	1627 (31.4)	1182 (39.9)	735 (24.8)
C3	2311 (44.6)	925 (17.9)	1781 (60.1)	928 (31.3)
C4	0 (0)	82 (1.6)	0 (0)	82 (2.8)
C5	0 (0)	3 (0.1)	0 (0)	4 (0.1)
C6	0 (0)	4 (0.1)	0 (0)	3 (0.1)

*LTF,* Long-term follow-up.

Data presented as number (%).

**Table III:** Univariate and multivariate analysis of primary and secondary outcomes

Variable	Univariate descriptive analysis			Multivariate logistic regression		
	Nonobese (n = 5183)	Obese (n = 2963)	*P* value	OR	95% CI	*P* value
Clinical outcome						
Change in VCSS	−3.35 ± 3.07	−3.29 ± 3.20	.408	0.968	0.85-1.10	.620
Change in PROs						
HASTI score	−6.62 ± 5.31	−7.24 ± 5.55	<.001	1.19	1.02-1.39	.026
Heaviness	−1.58 ± 1.57	−1.65 ± 1.60	.066	–	–	–
Achiness	−1.76 ± 1.56	−1.83 ± 1.57	.044	1.24	1.10-1.40	.001
Swelling	−1.33 ± 1.55	−1.49 ± 1.62	<.001	1.12	1.00-1.24	.043
Throbbing	−1.22 ± 1.49	−1.42 ± 1.56	<.001	1.38	1.20-1.60	<.001
Itching	−0.72 ± 1.42	−0.84 ± 1.53	<.001	1.25	1.13-1.38	<.001
Appearance	−1.56 ± 1.37	−1.47 ± 1.40	.007	0.98	0.88-1.10	.785
Work impact	−1.26 ± 1.42	−1.28 ± 1.50	.507	–	–	–
Complications						
Bleeding requiring intervention	4 (0.1)	4 (0.1)	.472	–	–	–
Blistering	17 (0.4)	10 (0.4)	1.000	–	–	–
DVT	34 (0.7)	32 (1.2)	.040	1.49	0.91-2.44	.116
Hematoma	23 (0.5)	12 (0.4)	.862	–	–	–
Paresthesia	104 (2.1)	64 (2.3)	.627	–	–	–
Pigmentation	42 (0.9)	13 (0.5)	.066	–	–	–
Superficial phlebitis	35 (0.7)	42 (1.5)	.001	1.82	1.15-2.87	.011
Treatment-induced ulcer	3 (0.1)	0 (0)	.558	–	–	–
Wound infection	15 (0.3)	8 (0.3)	1.000	–	–	–
EHIT	85 (1.7)	35 (1.3)	.125	–	–	–
Recanalization	56 (1.4)	33 (1.5)	.824	–	–	–

*CI,* Confidence interval; *DVT,* deep vein thrombosis; *EHIT,* endothermal heat-induced thrombosis; *HASTI,* heaviness, achiness, swelling, throbbing, itching; *OR,* odds ratio; *PROs,* patient-reported outcomes; *VCSS,* venous clinical severity score.

Data presented as mean ± standard deviation for continuous variables (assessed using Student’s *t* test and binomial logistic regression) or number (%) for categorical variables (evaluated using Fisher's exact test and binomial logistic regression).

Of the complication outcomes analyzed, obese limbs showed a larger incidence of superficial phlebitis (1.5% vs 0.7%; *P* = .001) and an increased incidence of postprocedure DVT formation (1.2% vs 0.7%; *P* = .040; Table III). No significant difference was found in any other analyzed follow-up complications or short-term recanalization. On multivariate analysis, the risk of superficial phlebitis (OR, 1.82; *P* = .011) remained statistically significant, although the increased risk of DVT formation did not (Table III).

**Fig 3:**
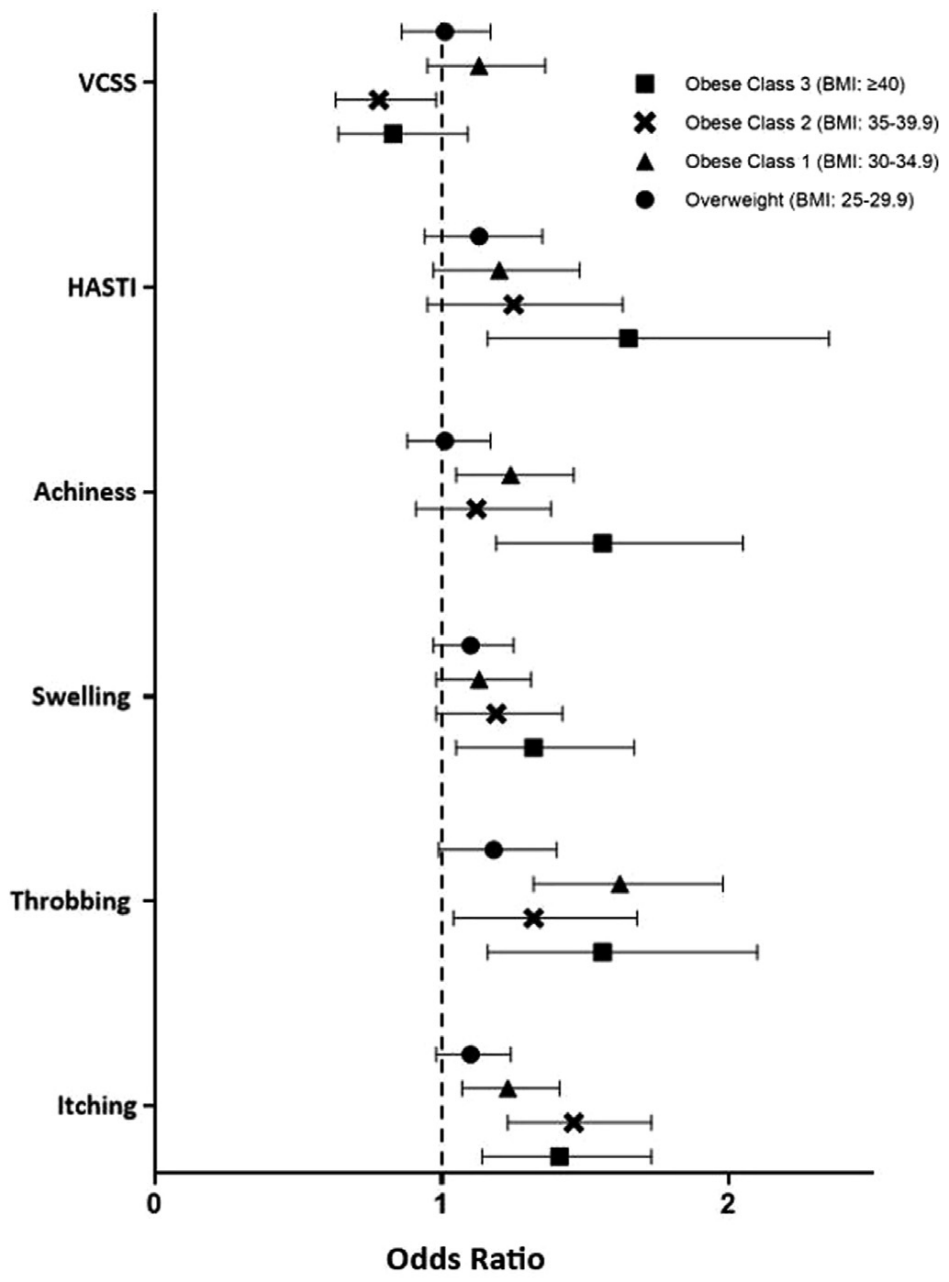
Odds ratios (ORs) and 95% confidence intervals (CIs) for multivariate analysis of primary outcomes and significant patient-reported outcomes (PROs), with obesity defined according to the five classes of the World Health Organization (WHO) classification. Normal weight (body mass index [BMI], <25 kg/m^2^) is used as the reference group.

Finally, postprocedural improvement using multiple logistic regressions with obesity defined by the WHO classification system were performed. Of the 8146 limbs, 2475 (30.4%) were classified as normal weight, 2708 (33.2%) as overweight, 1642 (20.2%) as class I obesity, 825 (10.1%) as class II obesity, and 496 (6.1%) as class III obesity. As demonstrated in Fig 3, the VCSS showed no significant trends or ORs, except for one significant OR (OR, 0.78; *P* = .029) demonstrating reduced improvement for class II obesity. There is a general increasing trend in ORs with increasing levels of obesity for the improvement in the HASTI score and the remaining variables of achiness, swelling, throbbing, and itching, indicating better improvement in their outcomes at higher classes of obesity. However, this is in the context of variable statistical significance (Fig 3). Superficial phlebitis demonstrated relatively increasing ORs with increasing levels of obesity in the context of the same variable significance, as evidenced by an OR of 1.71 (*P* = .144) for overweight limbs, an OR of 2.11 (*P* = .053) for class I obesity, an OR of 3.55 (*P* = .001) for class II obesity, and an OR of 2.27 (*P* = .096) class III obesity.

## Discussion

Although in many patients with C2 and C3 venous disease, endovenous treatment may be deferred or the disease managed conservatively, obese patients frequently present with more symptomatic disease and have an increased risk of disease progression.^2^ The presented data further supports this, with the observation that obese limbs presented with higher average scores for all PROs, except for limb appearance (Table I). Unfortunately, there is a relative paucity of research investigating the effects of obesity on endovenous therapy outcomes. One large study by Deol et al^19^ investigated obesity's impact on treatment, analyzing their center's institutional data via the percent change in the VCSS. They found that increasing levels of BMI resulted in less improvement after treatment.^19^ In the present study, the change in the VCSS was not found to be different between the obese and nonobese cohorts, with no significant identifiable trend among the WHO classification analysis. It is unclear why this study demonstrated no difference in the change in VCSS, especially because Deol et al^19^ found that decreased improvement remained even when stratifying by CEAP class, indicating their findings persist at CEAP classes C2 and C3. One explanation could be that they used a follow-up time of 6 months, and this analysis used the first reported follow-up after the procedure within 3 months. Perhaps the increased study time allowed for a better analysis of VCSS changes.

Interestingly, for C2 and C3 venous disease, the VCSS might not be the best method of assessing clinical improvement. Although the VCSS system surpasses the CEAP system in assessing changes in severity, the additional considerations of pain, inflammation, and compression therapy use, as well as accounting for the number and size of ulcers present, might not pertain to the disease severity found within patients in the C2 and C3 cohorts. Therefore, an analysis of more quality of life PROs, such as heaviness, achiness, swelling, throbbing, itching, appearance, and work impact might serve as a better measure of clinical effect. In this analysis, it was found that the limbs of obese patients show greater symptomatic improvement overall, as demonstrated by a greater improvement in the HASTI score and in almost all individual PROs, with better improvement associated with higher levels of obesity, although at various levels of significance (Fig 3). This is in contrast to previous research, as one study found that although obese patients show improvement in their symptoms, there is no difference in improvement in patients with an increasing BMI of ≥31 kg/m^2^ based on the CIVIQ-20 quality of life survey.^19^ Another study found that patients with a BMI >25 kg/m^2^ show worse quality of life scores, as assessed by the SF-36 (36-item short-form survey) after RFA compared with patients with a BMI of <25 kg/m^2^.^20^ However, the two studies used different methods of symptomatic assessment and failed to stratify by a staging method such as CEAP class, as was done in the present analysis, which was limited to CEAP C2 and C3 venous disease. This could suggest that obese patients with more severe disease do not experience the same improvement as those with milder disease. Conversely, the present findings demonstrating improved symptoms might simply be due to the analysis of the specifically targeted PROs included in the HASTI score rather than a full quality of life-type survey assessment. Regardless, it is important to highlight that in the present cohort, there was favorable symptomatic improvement in the limbs with higher classes of obesity compared with the nonobese limbs, although this was in the context of variable statistical significance at each obesity classification, and the results should be interpreted as such.

Perhaps the most concerning aspect of performing EVTA on obese populations is the rate of complications such as EHIT, DVT formation, and recanalization of the treated veins. However, no difference in the incidence of these complications was seen between the obese and nonobese cohorts. This is in line with previous research on EHIT and DVT formation after endovenous therapy, as studies investigating the incidence of venous thromboembolism after EVTA have shown obesity to not be a predictor of incidence.^21^, ^22^, ^23^ The literature on recanalization is less clear, such as one study demonstrating a BMI of >30 kg/m^2^ was associated with higher rates of recanalization. However, this was specifically in an EVLA combined with microphlebectomy population.^24^ Larger studies have shown BMI and obesity are not risk factors for recanalization.^25^^,^^26^ In this study, no difference was found in the incidence of early venous recanalization, although this study only analyzed the 3-month postprocedure data and did not consider longer term recanalization. An increased incidence was found of superficial phlebitis in obese limbs with higher classes of obesity associated with increasing odds of this complication. This finding is likely explained by the mechanism with which obesity is associated with CVI. Studies have found increased intra-abdominal pressure to correlate with decreased venous wall shear stress, which has been shown to instigate inflammatory reactions of the endothelial lining as a result of the disruption of physiologic flow, exacerbating CVI development.^27^, ^28^, ^29^, ^30^ This increased propensity for inflammatory reactions could theoretically lead to an increasing incidence of superficial phlebitis after treatment in obese populations, although biological studies are required to fully prove this theory.

The present study is not without limitations. This study is a retrospective analysis using previously collected data from the VQI. Many limbs in the VQI database had multiple veins treated during one procedure using a combination of techniques. The VQI lists other techniques as “surgery” but does not specify the surgery type for every procedure. Concomitant surgical procedures were accounted for as a whole in the multivariate analysis. However, this might not be granular enough to adequately account for how the use of combination techniques affect improvement. Other limitations are intuitive, including that only patients with both preprocedure and follow-up data were included, which excludes those lost to follow-up and could skew reporting. Additionally, with database analyses, a risk exists for misreporting of data, and with statistical model-based analyses, the risk exists of excluding important variables that might further explain the results. Finally, the use of self-reported outcomes is vulnerable to considerable variability and poor reproducibility among patients. As such, symptomatic improvement might not be due solely to thermal ablation interventions but to additional lifestyle factors unable to be captured in this database.

## Conclusions

To the best of our knowledge, this is one of the few studies to analyze EVTA in obese populations and the only study to emphasize more mild disease in the form of CEAP C2 and C3 venous disease. In the setting of improving endovenous treatment techniques and discussions surrounding more aggressive early intervention for patients with CVI, these findings become important in deciding whether obese patients should also be considered for earlier treatment of their disease process or if their body habitus would lead to minimal improvement. The results from the present study confirm that patients with C2 and C3 disease improve, both clinically and symptomatically after EVTA, regardless of obesity status. Obese patients report larger reductions in their perceived symptoms at early follow-up, with higher obesity classes weakly associated with better improvement in perceived symptoms. Although obesity has been associated with increased severity of venous disease symptoms, obese patients are able to derive significant relief after treatment during the short term and might provide greater relief of symptoms compared with nonobese patients when treated at more mild disease presentations. However, further prospective analyses are needed to explore the degree to which an earlier intervention should be offered to obese patients.

## Author contributions

Conception and design: ZZ, JG, MB, JE, AD, KN

Analysis and interpretation: ZZ, JG, BK, MB, KN

Data collection: ZZ, JG, GC

Writing the article: ZZ, JG, GC

Critical revision of the article: ZZ, JG, BK, MB, JE, AD, KN

Final approval of the article: ZZ, JG, GC, BK, MB, JE, AD, KN

Statistical analysis: ZZ, JG, KN

Obtained funding: Not applicable

Overall responsibility: JG

## Disclosures

None.

## Figures and Tables

**Supplementary Table I (online only):** 2020 revision of CEAP (clinical, etiology, anatomy, pathophysiology): summary of clinical classifications

CEAP class	Description
C0	No visible or palpable signs of venous disease
C1	Telangiectasias or reticular veins
C2	Varicose veins
C2r	Recurrent varicose veins
C3	Edema
C4	Changes in skin and subcutaneous tissue secondary to CVD
C4a	Pigmentation or eczema
C4b	Lipodermatosclerosis or atrophie blanche
C4c	Corona phlebectatica
C5	Healed
C6	Active venous ulcer
C6r	Recurrent active venous ulcer

*CVD,* Chronic venous disorder.

Data from Van der Velden et al.^26^

**Supplementary Table II (online only):** Analysis of outcomes from before procedure to initial follow-up for obese and nonobese cohorts

Variable	Nonobese (n = 5183)			Obese (n = 2963)		
	Preoperative	LTF	*P* value	Preoperative	LTF	*P* value
Clinical						
VCSS	6.42 ± 2.00	3.08 ± 2.57	<.001	6.80 ± 2.01	3.50 ± 2.75	<.001
PROs						
HASTI score	9.19 ± 4.83	2.57 ± 3.62	<.001	10.31 ± 5.21	3.07 ± 4.01	<.001
Heaviness	2.06 ± 1.46	0.48 ± 0.91	<.001	2.21 ± 1.51	0.56 ± 1.02	<.001
Achiness	2.52 ± 1.30	0.76 ± 1.06	<.001	2.67 ± 1.32	0.84 ± 1.15	<.001
Swelling	1.90 ± 1.51	0.57 ± 1.02	<.001	2.31 ± 1.57	0.82 ± 1.23	<.001
Throbbing	1.63 ± 1.43	0.41 ± 0.87	<.001	1.87 ± 1.51	0.45 ± 0.91	<.001
Itching	1.08 ± 1.37	0.37 ± 0.82	<.001	1.24 ± 1.46	0.40 ± 0.88	<.001
Appearance	2.50 ± 1.15	0.93 ± 1.02	<.001	2.40 ± 1.20	0.93 ± 1.05	<.001
Work impact	1.85 ± 1.24	0.60 ± 0.94	<.001	2.00 ± 1.28	0.72 ± 1.04	<.001

*HASTI,* Heaviness, achiness, swelling, throbbing, itching; *LTF,* long-term follow-up; *PROs,* patient-reported outcomes; *VCSS,* venous clinical severity score.

Data presented as mean ± standard deviation.
